# The landscape of basic gene therapy approaches in inherited retinal dystrophies

**DOI:** 10.3389/fopht.2023.1193595

**Published:** 2023-06-27

**Authors:** Jianhua Xia, Lei Gu, Qing Pan

**Affiliations:** The First Affiliated Hospital, Zhejiang University School of Medicine, Hangzhou, China

**Keywords:** gene therapy, inherited retinal dystrophies, adeno-associated virus, therapeutic window, animal model.

## Abstract

The study of gene therapies has been of particular interest in recent decades due to their promising potential to slow or even rescue the degeneration of the retina in inherited retinal dystrophies (IRDs). Here, we review the current approaches to gene therapy trials on IRDs, including the selection of animal models, therapeutic window, vectors and dosages. Mice are typically the first choice of animal models and recombinant adeno-associated virus (rAAV) of serotype 8 is the most common vector for loss-of-function IRDs. Furthermore, the therapeutic window should be considered to ensure efficacy before retinal degeneration occurs if possible, and dosages must be tailored to each approach.

## Introduction

Inherited retinal dystrophies (IRDs) are a group of diseases caused by genetic mutations those genes encode proteins in the retina. Affecting 1 in 3,000 people, these conditions can have a significant impact on study, work, and life patterns (1). While multiple methods have been attempted to ameliorate or delay the progression of IRDs, gene therapy has emerged as the most promising approach, particularly after the approval of voretigene neparvovec-rzyl (Luxturna) by US Food and Drug Administration (FDA) in 2017 (2). Luxturna is used to treat Leber congenital amaurosis (LCA), the most severe clinical type of IRD, which often results in rapid retinal degeneration from early childhood and is the most common cause of visual impairment in the first decades of life (3). This has spurred the development of gene editing engineering and intraocular gene therapies. Several genes associated with IRDs have been evaluated in animal/*in vitro* models and some have progressed to clinical trials, such as *GNAT2, CNGB3, CNGA3, GUCY2D, PED6B, RlBP1, MERTK, RPGR, ABCA4, MYO7A* and *RS1*, which have been reviewed in detail by Ku et al (4). A comprehensive understanding of these genes’ functions is essential for successful gene therapy and for making valid choices for gene clinical therapy approaches.

In this review, we discussed recent gene therapy experiences, focusing on the common choices of valid animal models, effective vector types, suitable therapeutic window, and dosage. We aimed to provide a resource to help researchers expedite their gene therapy designs.

## The choices of animal models

The use of animal disease models to simulate degenerative patterns closely related to human clinical findings is essential for researchers to investigate the underlying physiopathological processes. Prior to retinal genetic therapy trials, it is necessary to evaluate the expression level and location of target genes in each animal model, and it is preferable that these findings are comparable to those observed in human retinal tissues. In laboratory studies, animal models are widely used to assess the efficacy, safety and duration of treatment. However, due to species-specific barriers, some genes lack an ideal knock-down/out model. Kruczek et al. (5) recently reported that human stem cell-derived organoids can serve as a valid substitute for animal models. Nevertheless, organoids are unable to replicate immune responses and potential risks, making animal models irreplaceable for gene therapy tests.

The Swedish Bariard dog was the first reported in 1999 to spontaneously harbour a PRE65 mutation, leading to vision impairment resembling LCA (6). With breeding an inbred strain, subsequent gene therapy trials have demonstrated promising efficacy and safety profiles, paving the way for the *RPE65* clinical trials (7–9), thus ultimately led to the FDA-approved gene therapy drug Luxturna in 2017 (2). The canine eye is similar in volume to the human eye, making subretinal injection easier, and vision acuity tests and observation more accurate. Other canine models have been utilized for gene therapy approaches, such as those enabled by Clustered Regularly Interspaced Short Palindromic Repeats (CRISPR)/Cas9 technology (10). Despite these advantages, the higher breeding cost of canines, the lack of macular structure, and the presence of only two types of cone cells (S-cone and M/L-cone, the same as in rodents) have made canines a less popular choice for IRDs models.

Mice (mus musculus) is the most widely used animal model for ocular gene therapy due to its cellular retinal structure being closely related to that of humans. The development of CRISPR/Cas9 technology has enabled the generation of an array of target gene knock-down/out models in a relatively short period of time. Furthermore, numerous studies have reported the availability of multiple types of mutant mice models for various genes. Despite its advantages, mice is not a perfect retina model due to species differences between mice and humans, such as the lack of macular structure and only two cone cell types (S-cone and M/L-cone) suitable for nocturnal activity, resulting in either no retinal degeneration or a much slower manifestation than in humans (11). To assess the efficacy of gene therapy trials, functional tests of the retina such as electroretinogram (ERG) are employed, as well as the analysis of disease-associated depositions, such as N-retinyl-N-retinylidene ethanolamine (A2E) accumulation in Stargardt disease (11, 12). Thus, further testing in larger animal models is necessary to determine the curative effect and safety of each trial of target genes. In addition, the small size of the eye in mice imposes a limitation on subretinal injection, requiring skilled researchers to perform the procedure accurately. Typically, a 33-gauge needle is sufficient for mice subretinal injection, with an injection volume of 1μm.

Rat is another commonly used model for gene therapy, with a genome that is more closely related to that of humans than that of mice (13). Its retinal disease model can provide a more accurate presentation of rapid photoreceptor degeneration than mice, making it a preferred choice when mice disease models are not valid or when it is difficult to measure the pathophysiological changes associated with the target gene. Furthermore, rats possess a larger intraocular volume than mice, which makes subretinal injection easier.

Rabbit and non-human primates are commonly employed to assess safety and efficacy prior to clinical trials, likely due to the requirements of the USFDA for multiple species gene therapy trials prior to Phase I clinical trials (14). Rabbits possess a larger intraocular volume relative to humans and demonstrate closer immunological responses than rodents (15, 16). However, a disadvantage of this animal model for inherited retinal diseases is the lack of macular structure. Non-human primates, with the most similar genetic background to humans, and with macular structure and immunological responses, are the most suitable models for biodistribution and toxicological studies. The observation window of non-human privates for safety investigations of each therapy is longer, and the breeding cost of them is the highest among animal models.

There are other retinal disease animal models. Chicken and zebrafish are frequently employed in other ocular disease researches, such as chicken in myopia studies and zebrafish in photoreceptor function and embryo development (17, 18). However, due to structural and functional differences between mammals and these species, chicken and zebrafish are not recommended for IRDs gene therapy investigations (19, 20). Sheep have been used in one gene therapy investigation due to the discovery of a spontaneously mutated day blindness strain model in Israel (21). Large animal models such as sheep and dogs have a higher cone distribution than rodents, as well as a retinal structure and function more similar to that of humans. However, due to the lack of inbred strains and long breeding times with high costs, these species are not commonly chosen for ocular gene therapy (22).

## The choices for rAAV and promoter

For more than two decades, the recombinant adeno-associated virus (rAAV) vector has been widely used in gene augmentation therapies, particularly for genes with recessive inheritance patterns, considered as loss-of-function mutations (23). In contrast, for genes with dominant inheritance, gain-of-function mutations are more common, and a novel approach to interfere with mutated gene expression is RNA-based therapies such as antisense oligonucleotides (AONs). This approach has recently been reviewed in detail by Ku et al. and Girach et al. (4, 24).

While the rAAV vector has been widely used in gene augmentation therapy, it has limitations in cargo capacity with a maximum target gene size of 5kb and even smaller in self-complementary AAV (scAAV) of only 3.3kb. Thus, for gene therapy targeting larger coding proteins, equine infectious anemia virus (EIAV) or nanoparticles have been used as vectors (12, 25). Previous attempts to edit large target genes, such as *ABCA4* and *MYO7A*, into two pieces and remodel with dual rAAV vectors. Though the dual rAAV *ABCA4* treatment resulted in much lower efficient expression levels than EIAV therapies, Ferla et al. reported that the dual rAAV of *MYO4A* could achieve high mRNA expression in the visual pathway (26, 27). The limitations of current vectors and cargo capacity highlight the need for continued exploration and development of novel gene therapy approaches for IRDs.

To achieve higher transcription levels of the target genes in the target cells with limited off-target transcription, the appropriate rAAV capsid type and promoter need to be designed. Prior to undertaking any gene therapy, it is essential to have a complete understanding of the normal function and location of the target gene. Serotypes 2, 5, and 8 of the rAAV capsid are commonly used with high retinal tissue affinity, serotype 8 being the most popular one (4). Other newly reported rAAVs, such as AAV9 and AAV8(Y733F), have also demonstrated satisfied expression effects (28, 29). Self-complementary AAV (scAAV) has been shown to have a higher transduction efficiency than single-stranded AAV (ssAAV) in many different trials, making it the default editing structure nowadays (28, 30, 31).

Promoter, placed in the rAAV DNA strand before the target gene coding sequence, is critical to achieve more effective expression level. Suitable promoter types are chosen to be specifically expressed in the target cell types. For example, the widely used promoter of the CMV gene can transfect many types of cells, including photoreceptors and retinal pigmentation epithelium (RPE) cells, whereas the human opsin promoter can successfully and specifically transfect mice photoreceptors (10, 11). Some investigators have used the human promoter of the target gene directly (9, 30). To determine the best combination of vector type and promoter, pre-testing in animal models is necessary (30).

Gene augmentation therapy using AAV vectors cannot resolve all loss-of-function situations even with appropriate target gene size. AAV-mediated *Tulp1* gene supplement therapy has provided minimal benefits in *Tulp1* knock-out mice model, suggesting that fine-tuning the supplement strategy is necessary (32). Therefore, the combination of non-specific strategies with gene therapy may yield better outcomes in such situations.

## The choices of therapeutic windows and dosage

Researchers are constantly pondering whether there is an optimal therapeutic period. Various research designs and opinions exist for different target genes. Narfström et al. conducted a gene therapy trial on Swedish Bariard dogs ranging from 4 to 30 months old for *RPE65* gene therapy and found that younger dogs had better ERG recovery than elder dogs (8). Conversely, some trials reported no preservation in elder disease models, such as *SPATA7* gene therapy (33). Cepko and Vandenberghe proposed the existence of a “point of no return” in gene therapy, suggesting that the rescue of gene therapy should occur before severe retinal degeneration happens (34). The “point of no return” is dependent on the pathophysiologic mechanism of each gene, where some genes cause early retinal degeneration while others are relatively stable. Langlo et al. stated that in some stable IRDs, such as *CNGB3*-caused achromatopsia, theoretically there are no treatment period limits (35). The combination of neuron protection therapy or other supplement of factors may help to improve the curative effect compared to rAAV rescue alone and delay the “point of no return”.

Recent research has emphasized the importance of a better curative effect, given the imperfect long-term prognosis for Luxturna treatment, where retinal degeneration continues after the treatment of more than one decade (3). Our group investigated different rescue times in *LCA* model *Rdh12* ^-/-^ mice and concluded that earlier gene therapy had more benefits, even before the occurrence of retinal degenerations (11). Earlier restock of normal protein can successfully block the abnormal molecular/biochemical procedures and make the rescued retina more resistant to light-induced damage, resulting in a better curative effect. It is essential to determine the best therapeutic period to benefit patients, which may even be before the pathologic presentation in the retina. Moreover, long-term observation is necessary to determine whether the expression level of target genes declines after injection.

Dosage testing in an animal model with gradient is also crucial. The titer is usually designed from 10^6^ vector genomes (vg)/ml to 10^11^ vg/ml. A low titer may have no effective expressions of target genes in degenerated cells, while a higher titer could have higher expression levels but severe side effects, such as intraocular inflammation and uveitis (10, 11). It is reported that the expression level cannot increase linearly with increasing dosage but reaches a plateau after a certain point. Researchers need to find a suitable dosage to balance the expression level and the dosage-associated inflammation. We further hypothesize that there may be different levels of valid dosage for preventative therapy and rescuing therapy (31). Although Bian et al. utilized the same dosage in preventative and rescuing therapy, it is worth investigating whether there is a difference between these two intervention windows regarding the relationship of dosage and curative effect, thus helping to achieve better treatment outcomes (11).

## Summary

In this article, we provide an overview of several key factors in ocular gene therapy for IRDs. The use of target gene knock-down/out mice as an animal disease model has been widely adopted for initiating gene therapy. The choice of vectors is crucial for effective gene therapy, with rAAV being the most commonly used vector. However, novel vectors such as nanoparticles have shown promise for improving the transduction efficacy. In addition, the use of optimized promoters has been shown to enhance gene expression in the retina. The therapeutic window for gene therapy could be expanded to cover both the preventative and rescuing periods of disease progression. Accurate dosage of gene therapy is essential and should be tailored to the specific disease process or level of retinal degeneration to achieve an effective rescue. Finally, the development of non-specific strategies combined with gene therapy may offer new possibilities for expanding the potency of treatment in IRDs.

## Author contributions

JX generated all the researches’ data. LG wrote the main manuscript. QP supervised this manuscript. All authors contributed to the article and approved the submitted version.
